# Prognostic value of lymphocyte to monocyte ratio for cervical cancer: a systematic review and meta-analysis

**DOI:** 10.7717/peerj.21337

**Published:** 2026-05-27

**Authors:** Guanjun Zhao, Masmuha Marshed Al Ramadhan, Yifang Zhang

**Affiliations:** Department of Obstetrics and Gynecology, The Second Affiliated Hospital of Shandong First Medical University, Shandong First Medical University & Shandong Academy of Medical Sciences, Taian, Shandong Province, China

**Keywords:** Prognosis, Lymphocyte-to-monocyte ratio, Cervical cancer, Meta-analysis, Systematic review

## Abstract

**Background:**

Systemic inflammatory biomarkers have been associated with outcomes across multiple malignancies, including cervical cancer. The prognostic value of the pretreatment lymphocyte-to-monocyte ratio (LMR) in cervical cancer remains inconsistent across published studies. We therefore performed a systematic review and meta-analysis to clarify the association between pretreatment LMR and survival outcomes in cervical cancer.

**Methods:**

PubMed, Web of Science, Embase, and the Cochrane Library were searched from database inception to November 14, 2025. Studies evaluating pretreatment LMR in relation to survival outcomes in cervical cancer were included. Hazard ratios (HRs) with 95% confidence intervals (CIs) were pooled for overall survival (OS) and progression-free survival (PFS). Subgroup, sensitivity, meta-regression, and publication-bias analyses were also performed.

**Results:**

Eighteen studies comprising 22 independent cohorts (*n* = 5,127) were included. Low pretreatment LMR was associated with poorer OS in both univariable (HR = 1.94, 95% CI [1.58–2.38]) and multivariable analyses (HR = 1.66, 95% CI [1.40–1.96]), and with poorer PFS in both univariable (HR = 2.26, 95% CI [1.77–2.90]) and multivariable analyses (HR = 2.07, 95% CI [1.47–2.90]). In multivariable subgroup analyses, the adverse OS association remained evident in patients aged <50 years, in studies using an LMR cut-off <3.85, and in cohorts treated primarily with surgery. Meta-regression further indicated that study-specific LMR cut-off values were significantly associated with OS effect estimates.

**Conclusions:**

Current evidence suggests that a low pretreatment LMR is significantly associated with poorer OS and PFS in cervical cancer. Pretreatment LMR may therefore represent an accessible and cost-effective biomarker for pretreatment risk stratification. Nevertheless, prospective studies are warranted to validate clinically meaningful cut-off values, standardize analytical approaches, and further define the clinical utility of LMR in cervical cancer.

## Introduction

Cervical cancer remains a major global health burden, ranking among the most commonly diagnosed cancers and leading causes of cancer-related death in women. In 2022, an estimated 604,000 new cases and 342,000 deaths occurred worldwide (Bray et al., 2024). Persistent infection with high-risk human papillomavirus (hrHPV) is the primary etiological factor for cervical cancer, with HPV16 and HPV18 accounting for the majority of cases (Crosbie et al., 2013; Voelker, 2023). Although population-level interventions—HPV vaccination, screening, and timely treatment of precancerous lesions—have improved early detection and outcomes, prognosis remains heterogeneous, particularly for patients with locally advanced disease who are commonly treated with radiotherapy and/or chemotherapy and experience higher recurrence rates than those diagnosed at early stages (Abu-Rustum et al., 2023; Xu et al., 2025). Established prognostic factors (*e.g.*, tumor size, nodal status, histology and grade, and lymphovascular space invasion) are frequently determined postoperatively and therefore have limited utility for initial risk stratification and treatment planning (Abu-Rustum et al., 2023). Consequently, there is a clinical need for accessible and easily obtainable biomarkers that can support pretreatment risk assessment and guide follow-up strategies.

Inflammation contributes to tumor initiation and progression through dynamic interactions among cancer cells, stromal components, and immune cells within the tumor microenvironment (Greten & Grivennikov, 2019; Denk & Greten, 2022). Consistent with this biology, peripheral blood-based inflammatory indices derived from routine complete blood counts—such as the neutrophil-to-lymphocyte ratio (NLR), platelet-to-lymphocyte ratio (PLR), and lymphocyte-to-monocyte ratio (LMR)—have been investigated as prognostic markers across multiple malignancies and in gynecologic cancers (Diem et al., 2017; Dai et al., 2019; Tan et al., 2024; Zhang et al., 2023). In cervical cancer, growing evidence suggests that these indices may correlate with survival outcomes (Guo et al., 2023b; Koca et al., 2025). However, the specific prognostic value of pretreatment LMR remains uncertain, and prior evidence syntheses were limited by the number of available studies (Han et al., 2021). Therefore, we conducted an updated systematic review and meta-analysis to clarify the association between pretreatment LMR and survival outcomes in patients with cervical cancer.

## Materials & Methods

### Study registration and reporting

This systematic review and meta-analysis was reported in accordance with the Preferred Reporting Items for Systematic Reviews and Meta-Analyses (PRISMA) 2020 statement (Page et al., 2021). The review protocol was prospectively registered in the International Prospective Register of Systematic Reviews (PROSPERO; registration number CRD420251022134).

### Search strategy

Two reviewers (G. Zhao and M. Al Ramadhan) independently searched PubMed, Embase, Web of Science, and the Cochrane Library from database inception to November 14, 2025. The search strategy combined controlled vocabulary (*e.g.*, MeSH/Emtree) and free-text terms related to “lymphocyte-to-monocyte ratio/LMR” and “cervical cancer.” Reference lists of eligible articles and relevant reviews were manually screened to identify additional studies. The full electronic search strategies for each database are provided in the Supplementary Material.

### Eligibility criteria

Studies were included if they: (1) enrolled women with pathologically confirmed cervical cancer; (2) evaluated the association between pretreatment LMR and survival outcomes; (3) reported hazard ratios (HRs) with 95% confidence intervals (CIs) for overall survival (OS) and/or progression-free survival (PFS), or provided sufficient data to derive them; and (4) specified a cut-off defining low *versus* high LMR. We excluded: comments, letters, guidelines, narrative reviews, case reports, conference abstracts, and meeting summaries; animal studies; studies with duplicated/incomplete data; and studies with overlapping patient populations (in which case, the most informative or most recent dataset was retained). Only studies published in English were included.

### Data extraction

Records were imported into EndNote (version 20), and duplicates were removed. Two reviewers independently screened titles and abstracts, followed by full-text assessment against the eligibility criteria. Using a standardized extraction form, the same reviewers independently extracted the following data: first author, publication year, country, study period, sample size, age, FIGO stage/grade (as reported), histology, primary treatment, LMR cut-off, follow-up duration, survival outcomes (OS/PFS), analysis type (univariable or multivariable), and HRs with 95% CIs. To ensure a consistent direction of effect across studies, HRs were harmonized such that HR >1 indicated poorer survival in patients with low pretreatment LMR relative to those with high pretreatment LMR. We additionally extracted methodological details related to cut-off derivation, including whether the LMR threshold was median-based, ROC-/Youden-based, or otherwise data-driven. We also recorded whether formal internal or external validation was reported, the timing of blood sampling relative to treatment initiation, whether LMR was assessed only once before treatment or serially during follow-up, and the covariates included in multivariable models whenever available. Detailed methodological extraction items related to cut-off derivation, blood sampling, serial assessment, and multivariable model handling are summarized in Table S2. Across the included studies, these methodological features were heterogeneous, and formal validation of data-driven cut-offs was infrequently reported. Disagreements between the two reviewers were resolved through discussion with a third reviewer until consensus was reached. OS was defined as the primary outcome and PFS as the secondary outcome.

### Quality assessment

Study quality was assessed using the Newcastle-Ottawa Scale (NOS). Two reviewers independently scored each study, with scores ≥6 (out of 9) considered high quality. Any discrepancies were resolved by discussion.

### Statistical analysis

Hazard ratios (HRs) with 95% confidence intervals (CIs) were pooled as summary effect estimates. Where both univariable and multivariable HRs were available, multivariable estimates were prioritized for the primary meta-analysis. Statistical heterogeneity was assessed using Cochran’s *Q* test and the I^2^ statistic. A fixed-effect model was used when heterogeneity was low (I^2^ <  50%); otherwise, a random-effects model was applied. Prespecified subgroup analyses were conducted according to age (<50 *vs* ≥50 years), histology (squamous cell carcinoma *vs* mixed/other), primary treatment (surgery *vs* RT/CRT/NACT), and LMR cut-off (<3.85 *vs* ≥3.85). The subgroup threshold of 3.85 corresponded to the median study-specific LMR cut-off across the included cohorts. Accordingly, studies were stratified into <3.85 and ≥3.85 groups for subgroup analysis. This threshold was used as a pragmatic study-level stratification value rather than a universally validated biological or clinical cut-off. Sensitivity analyses were performed using a leave-one-out approach. Meta-regression using the study-specific LMR cut-off value as the moderator was conducted for OS analyses to explore potential sources of heterogeneity and to examine whether pooled effect estimates depended on the cut-off value. Publication bias was assessed using funnel plots and Egger’s test, with results interpreted cautiously when the number of contributing studies was limited. All meta-analyses were performed using Review Manager (RevMan) 5.4 and Stata 18. A two-sided *P* value <0.05 was considered statistically significant unless otherwise specified.

## Results

### Search results

**Figure 1:**
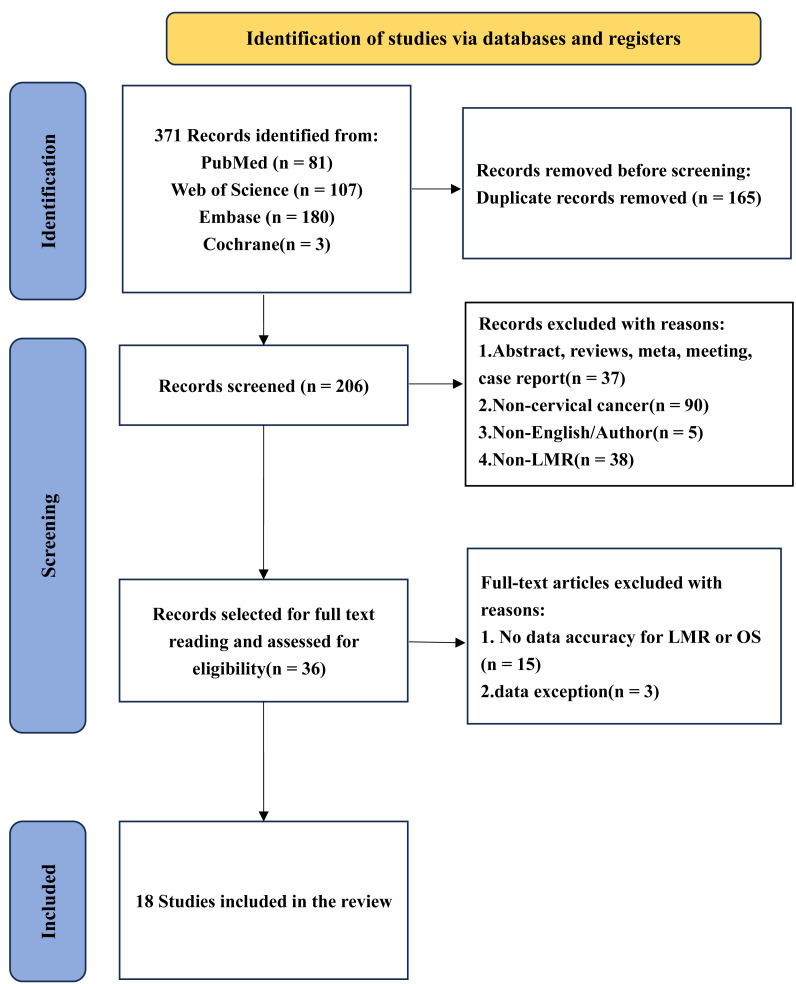
Identification of studies *via* databases and registers.

As shown in Fig. 1, the database search identified 371 records (PubMed, *n* = 81; Web of Science, *n* = 107; Embase, *n* = 180; Cochrane, *n* = 3). After removing 165 duplicate records, 206 records remained for title and abstract screening. At this stage, 170 records were excluded for predefined reasons (abstract/review/meta-analysis/meeting report/case report, *n* = 37; non-cervical cancer, *n* = 90; non-English, *n* = 5; non-LMR, *n* = 38), leaving 36 reports for full-text assessment. After full-text review, 18 reports were excluded (insufficient data to extract or calculate effect estimates for LMR and OS, *n* = 15; data issues/overlapping data, *n* = 3). Finally, 18 studies were included in the meta-analysis. The PRISMA flow diagram is presented in Fig. 1.

### Characteristics of included studies

The characteristics of the included studies are summarized in Table 1. Eighteen eligible studies (Ayhan et al., 2022; Chao et al., 2020; Chen et al., 2015; Chen et al., 2024; Cheng et al., 2022; Deng et al., 2021; Gao et al., 2024; Guo et al., 2023a; Guo et al., 2023b; Hao et al., 2025; Huang et al., 2019; Jia et al., 2025; Koca et al., 2025; Kumar et al., 2024; Li et al., 2021; Liu et al., 2022; Wang et al., 2023; Xu et al., 2021), comprising 22 independent cohorts and a total of 5,127 patients, were included in this meta-analysis. All studies had retrospective cohort designs and were published between 2015 and 2025. Most studies were conducted in China (*n* = 15), with the remaining studies from Turkey (*n* = 2) and India (*n* = 1). Four articles contributed two analytically independent cohorts each, yielding 22 cohorts in total. Regarding treatment setting, seven studies mainly involved early-stage patients treated with surgery-based approaches, four focused on locally advanced disease managed primarily with chemoradiotherapy, six enrolled mixed-stage or non-surgical populations, and one evaluated combination immunotherapy. Histologic composition was predominantly squamous cell carcinoma, although several cohorts also included adenocarcinoma or mixed histologies. Cohort sample sizes ranged from 70 to 1,051 patients, and the reported LMR cut-off values varied considerably, ranging from 2.44 to 12.25. Study quality was generally acceptable, with Newcastle-Ottawa Scale scores ranging from 6 to 8 (Table S1).

**Table 1:** Baseline characteristics of included studies.

Author	Year	Region	Sample size	Mean/ median age	FIGO stage	Treatment	Histological type	Follow-up (month) Median (range)	LMR	Survival outcome	Survival analysis	NOS
Koca et al. (2025)	2025	Turkey	90	NA	I–IV	CRT	SCC/AC/others	36.2 (4.9–117.4)	3.33	OS/PFS	U/M	7
Jia et al. (2025)	2025	China	157	55	IIIB-IVA	CRT	SCC	35 (3–96)	6.2	OS/PFS	U	8
Hao et al. (2025)	2025	China	99	60	I–IV	RT	SCC	33	3.5	OS	U	7
Chen et al. (2024)	2024	China	138	60.1	IB-IVA	CRT	SCC/AC/others	33.8 (7.2–79.3)	3.85	OS/PFS	U	7
Kumar et al. (2024)	2024	India	1,051	50	IB2-IVA	CRT	SCC/AC/others	69 (3–203)	4.41	OS	U	8
Wang et al. (2023)	2023	China	178	53.85	II–III	RT	SCC/AC	NA	2.44	OS/PFS	U/M	8
Gao et al. (2024)	2024	China	186	47.11	IIIC1p	radical operation+CRT	SCC/AC/others	51.1 (30–91)	3.85	OS	U/M	7
Guo et al. (2023b)	2023	China	109	53.95	I-IIA	radical operation	SCC	NA	4.17	OS	U	8
Guo et al. (2023a)	2023	China	196	52	IIB-IIIC	CRT	SCC/AC	64.8	4.04	OS	U/M	7
Liu et al. (2022) (1)	2022	China	133	51	IB2-IIB	NACT+radical operation	SCC	98 (4–156)	12.25	OS/PFS	U	8
Liu et al. (2022) (2)	2022	China	77	48	IB2-IIB	NACT+radical operation	SCC	84.6 (13–106)	12.25	OS	U	8
Cheng et al. (2022)	2022	China	70	51	NA	Combination Immunotherapy	SCC/AC/others	NA	3.45	PFS	U	6
Ayhan et al. (2022)	2022	Turkey	163	49	IA-IIIC	radical operation ±RT/CT	SCC/AC/others	NA	3.66	OS	U	8
Xu et al. (2021)	2021	China	264	47	IB-IIA	radical operation	SCC/AC/others	44 (3–72)	4.1	OS	U/M	8
Li et al. (2021)	2021	China	260	51	IIB	NACT ±RT	SCC/AC/others	47.3 (2.63–101.4)	3.85	OS/PFS	U/M	8
Deng et al. (2021) (1)	2021	China	283	47	IA-IIA	radical operation±RT	SCC	72 (4–129)	3.7	OS	U/M	7
Deng et al. (2021) (2)	2021	China	125	43	IA-IIA	radical operation±RT	SCC	47 (3–120)	3.7	OS	U/M	7
Chao et al. (2020) (1)	2020	China	441	42	IA-IIA	radical operation ±RT/CT	SCC	67 (6–129)	3.45	OS	U/M	8
Chao et al. (2020) (2)	2020	China	164	NA	IA-IIA	radical operation ±RT/CT	SCC	NA	3.45	OS	U/M	8
Huang et al. (2019) (1)	2019	China	328	45	IA-IIA	radical operation	SCC	47 (3–129)	3.85	OS	U/M	8
Huang et al. (2019) (2)	2019	China	130	44	IA-IIA	radical operation	SCC	47 (3–120)	3.85	OS	U/M	8
Chen et al. (2015)	2015	China	485	45	IB1-IIA	radical operation ±RT/CT	SCC/AC/others	75 (10-118)	2.87	OS	U/M	7

**Notes.**

CRT chemoradiotherapy RT Radiotherapy NACT neoadjuvant chemotherapy CT chemotherapy SCC Squamous cell carcinoma AC Adenocarcinoma OS overall survival PFS progression-free survival LMR lymphocyte-to-monocyte ratio NOS Newcastle–Ottawa Scale NA Not available U Univariate analysis M Multivariate analysis

(1)(2) Two independent cohorts in the same study.

### Association between LMR and overall survival

#### Univariable OS

**Figure 2:**
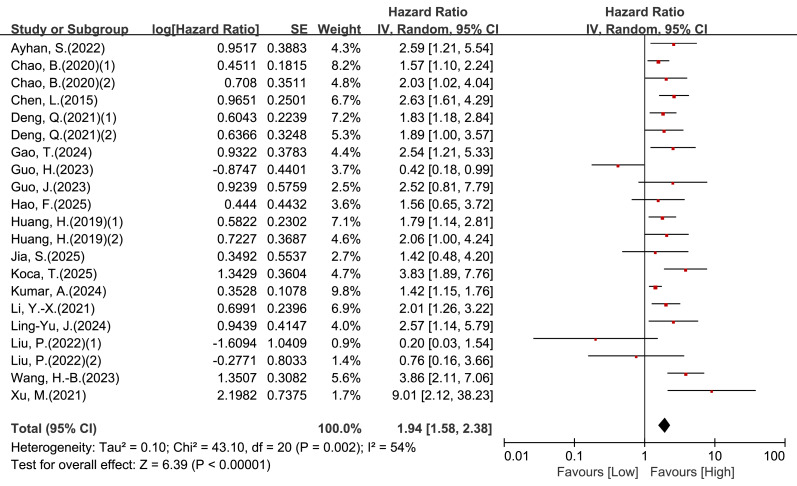
Forest plot of association between LMR and OS in patients with cervical cancer (univariate analysis).

Twenty-one cohorts reported univariable associations between LMR and OS. Moderate heterogeneity was observed (I^2^ = 54%, *P* = 0.002); therefore, a random-effects model was applied. The pooled estimate showed that low LMR was associated with worse OS (HR = 1.94, 95% CI [1.58–2.38], *P* < 0.00001; Fig. 2 and Table 2).

In subgroup analyses stratified by age category, LMR cut-off value, histology, and primary treatment, the direction of the association was generally consistent, with low LMR remaining a poor prognostic factor in cohorts treated primarily with surgery and in those treated with radiotherapy/chemoradiotherapy (Table 2).

#### Multivariable OS

**Figure 3:**
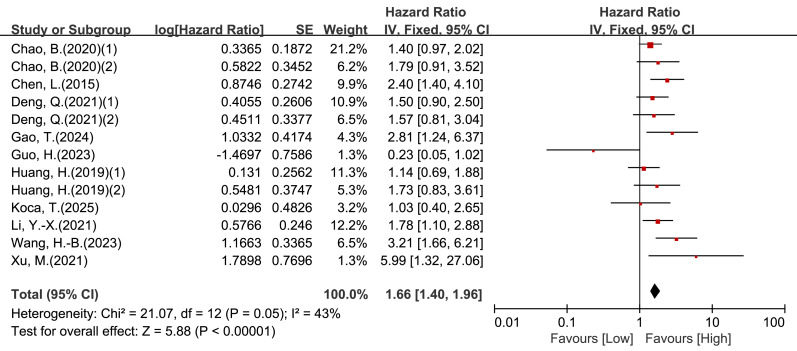
Forest plot of association between LMR and OS in patients with cervical cancer (multivariate analysis).

Ten studies comprising 13 cohorts reported multivariable OS estimates. Heterogeneity was low-to-moderate (I^2^ = 43%, *P* = 0.05), and a fixed-effect model was used. Low pretreatment LMR remained significantly associated with inferior OS (HR = 1.66, 95% CI [1.40–1.96], *P* < 0.00001; Fig. 3). In subgroup analyses (Table 2), significant associations persisted in several multivariable subgroups, including patients aged <50 years, studies using an LMR cut-off <3.85, surgery-based cohorts, and both histologic subgroup categories.

**Table 2:** Subgroup analysis of prognostic role of LMR for overall survival in patients with cervical cancer.

Subgroup	OS-Univariate				OS-Multivariate			
	Number of studies	HR (95% CI)	*P*	I^2^	Number of studies	HR (95% CI)	*P*	I^2^
Total	21	1.94 [1.58, 2.38]	<0.00001	54%	13	1.66 [1.40, 1.96]	<0.00001	43%
Mean/median age	
<50	10	1.96 [1.64, 2.34]	<0.00001	8%	8	1.68 [1.39, 2.04]	<0.00001	33%
≥50	9	1.60 [1.06, 2.41]	0.02	69%	3	1.40 [0.51, 3.81]	0.51	80%
unknown	2	2.77 [1.69, 4.53]	<0.00001	37%	2	1.48 [0.86, 2.57]	0.16	0%
Cut-off value	
<3.85	9	2.13 [1.77, 2.56]	<0.00001	29%	7	1.71 [1.38, 2.11]	<0.00001	21%
≥3.85	12	1.67 [1.21, 2.29]	0.002	58%	6	1.60 [0.96, 2.69]	0.07	62%
Histological type	
SCC	11	1.71 [1.42, 2.07]	<0.00001	0%	6	1.44 [1.16, 1.79]	0.0009	0%
SCC/others	10	2.27 [1.56, 3.29]	<0.0001	75%	7	1.96 [1.21, 3.15]	0.006	61%
Primary treatment	
Surgery	11	2.00 [1.68, 2.37]	<0.00001	0%	9	1.64 [1.35, 1.98]	<0.00001	15%
CRT/RT/NACT	10	1.64 [1.08, 2.47]	0.02	72%	4	1.34 [0.61, 2.94]	0.46	74%

**Notes.**

CRT chemoradiotherapy RT radiotherapy NACT neoadjuvant chemotherapy SCC squamous cell carcinoma

### Association between LMR and progression-free survival

**Figure 4:**
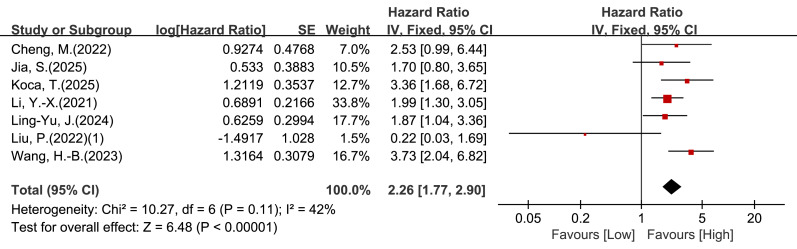
Forest plot of association between LMR and PFS in patients with cervical cancer (univariate analysis).

Seven studies reported univariable associations between LMR and PFS. Given moderate heterogeneity (I^2^ = 42%, *P* = 0.11), a fixed-effect model was applied. Low LMR was associated with worse PFS (HR = 2.26, 95% CI [1.77–2.90], *P* < 0.00001; Fig. 4).

**Figure 5:**
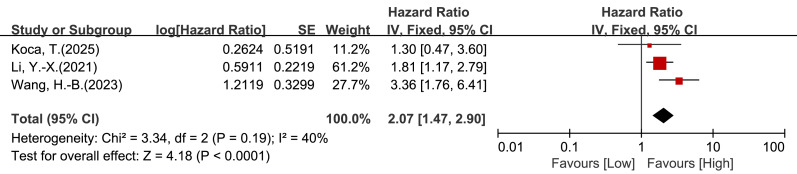
Forest plot of association between LMR and PFS in patients with cervical cancer (multivariate analysis).

Three studies (*n* = 528) provided multivariable PFS estimates. Heterogeneity was low (I^2^ = 40%, *P* = 0.19), and a fixed-effect model was used. The pooled multivariable result also showed an association between low LMR and poorer PFS (HR = 2.07, 95% CI [1.47–2.90], *P* < 0.0001; Fig. 5). Because only three studies contributed to this analysis, the result should be interpreted cautiously.

### Sensitivity analysis

**Figure 6:**
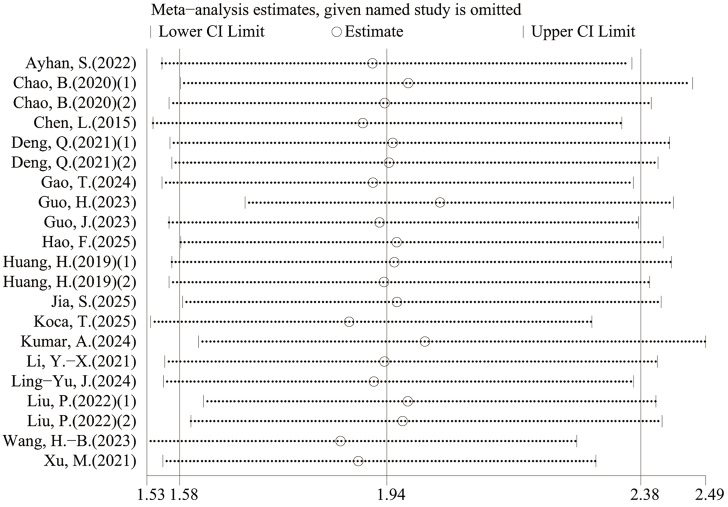
Sensitivity analysis (OS-univariable).

**Figure 7:**
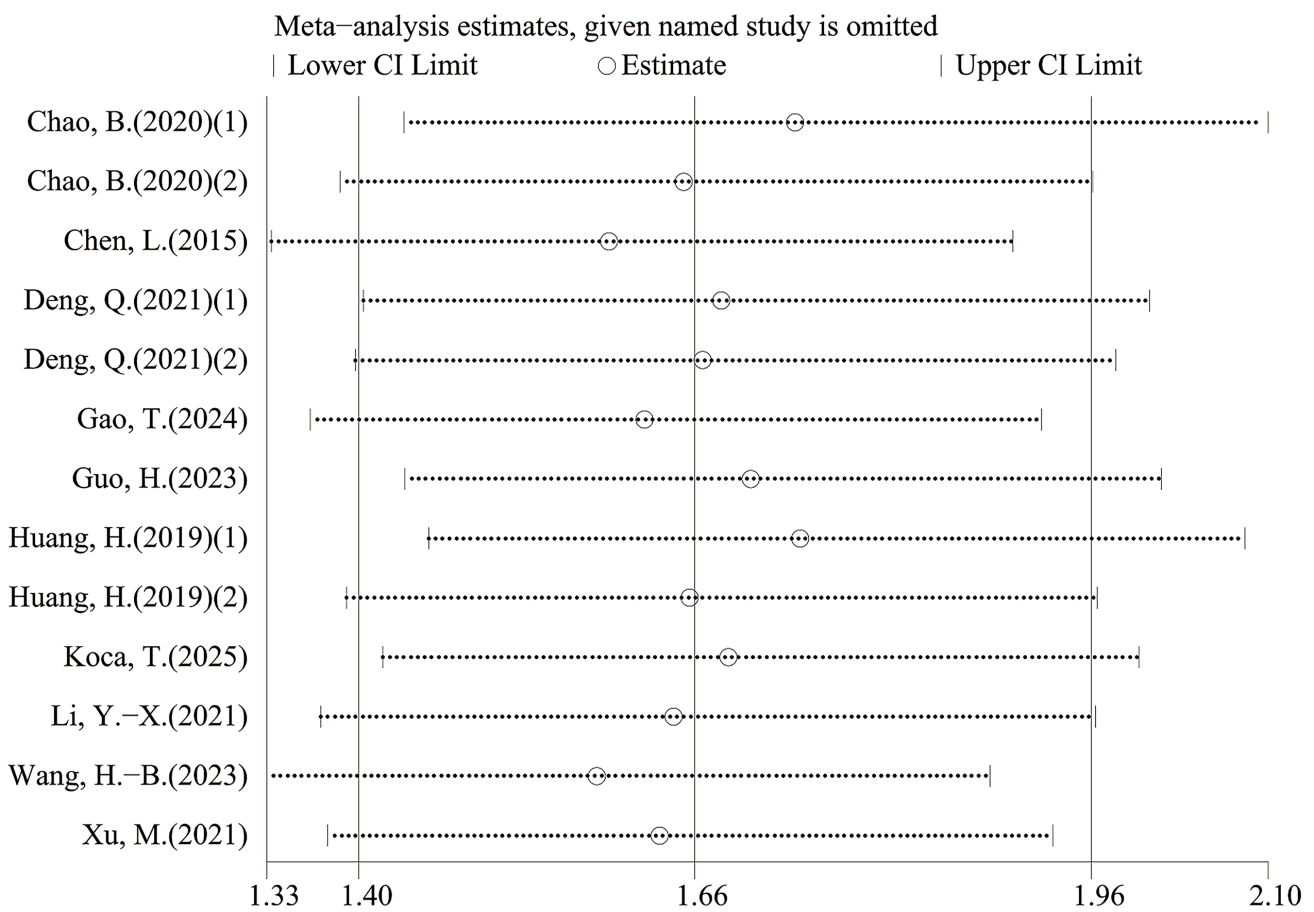
Sensitivity analysis (OS- multivariable).

**Figure 8:**
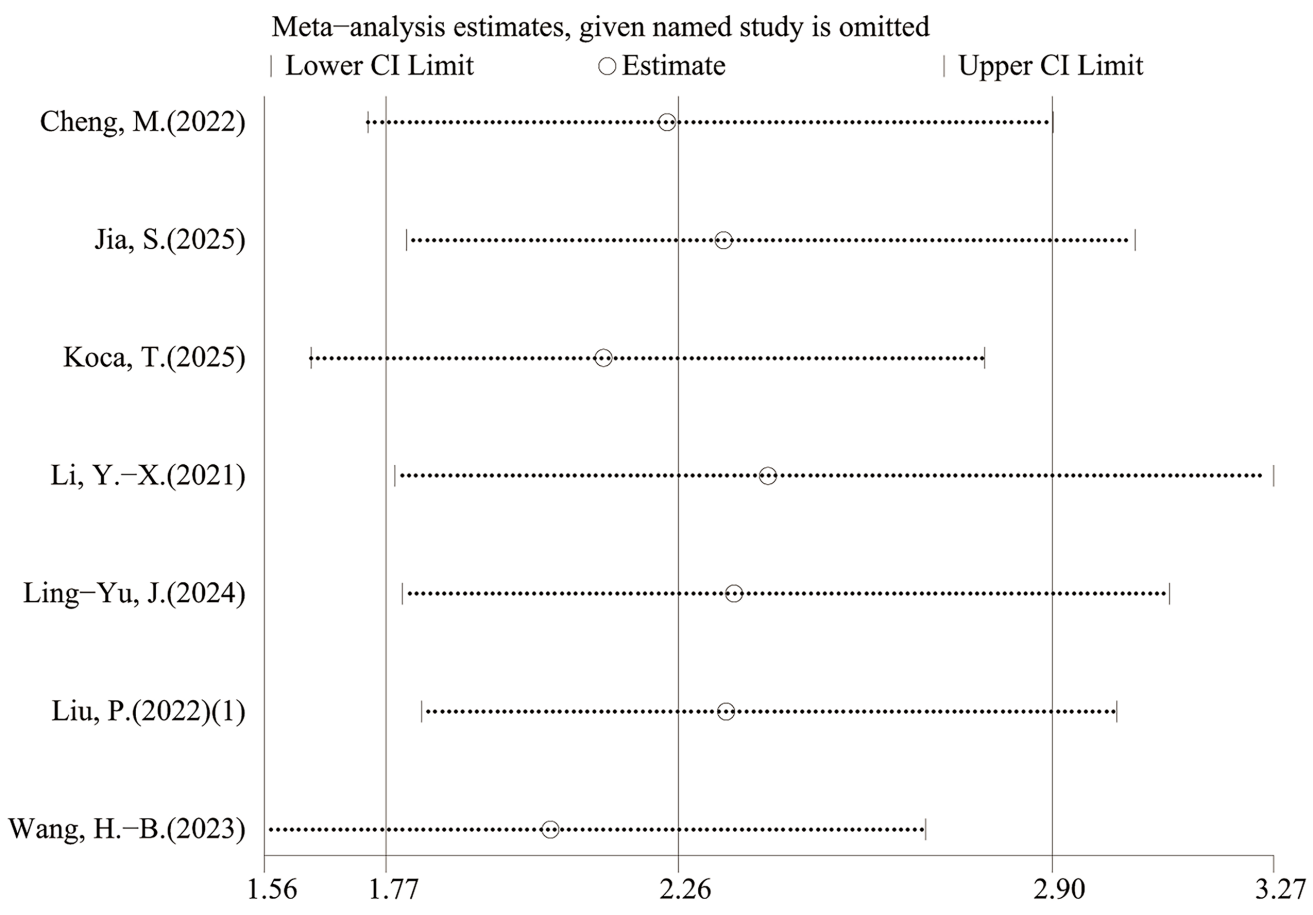
Sensitivity analysis (PFS-univariable).

**Figure 9:**
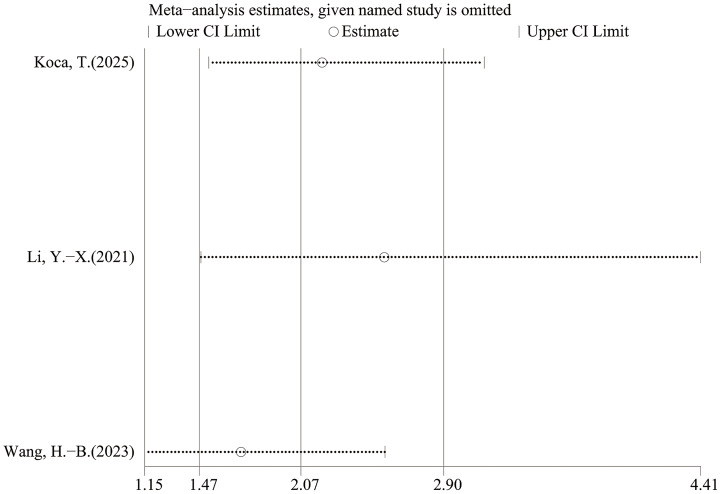
Sensitivity analysis (PFS- multivariable).

Leave-one-out sensitivity analyses indicated that the pooled estimates for OS and PFS were not materially changed by exclusion of any single study, suggesting that the results were robust (Figs. 6–9).

### Meta-regression analysis

To explore potential sources of heterogeneity, we performed meta-regression using the study-specific LMR cut-off value as the moderator. The cut-off value was significantly associated with effect size in both the univariable OS analysis (coefficient = −0.216, *P* = 0.001) and the multivariable OS analysis (coefficient = −0.440, *P* = 0.035), suggesting that higher LMR cut-off values were associated with smaller OS effect estimates (Figs. S1 and S2).

### Publication bias

Funnel plot inspection suggested no marked asymmetry for OS (Figs. S3–S4). Egger’s regression test showed no evidence of small-study effects for OS in the univariable analysis (*P* = 0.368) or multivariable analysis (*P* = 0.778) (Fig. S7). For PFS, the number of studies was small (univariable, *n* = 7; multivariable, *n* = 3), and assessments of publication bias should therefore be interpreted cautiously.

## Discussion

Cervical cancer remains a major cause of cancer-related mortality among women worldwide, and clinical outcomes vary substantially across stages and treatment settings despite advances in screening and therapy (Bray et al., 2024; Xu et al., 2025). This heterogeneity highlights the need for accessible biomarkers that can support pretreatment risk stratification and inform follow-up intensity. Peripheral blood-based inflammatory indices have attracted interest because they are inexpensive, readily available, and may reflect host immune-inflammatory status (Han et al., 2021).

In this systematic review and meta-analysis of 18 studies (22 cohorts; *n* = 5,127), low pretreatment LMR was associated with poorer overall survival (OS) and progression-free survival (PFS) in cervical cancer. In multivariable analyses, low LMR was associated with worse OS (HR = 1.66, 95% CI [1.40–1.96], *P* < 0.00001; Fig. 3) and worse PFS (HR = 2.07, 95% CI [1.47–2.90], *P* < 0.0001; Fig. 5). These findings are broadly consistent with evidence from other solid tumors, where lower LMR has been linked to unfavorable outcomes, including glioma and endometrial cancer (Wang, Xu & Zhang, 2023; Huang, Yang & Liu, 2025). Collectively, the results support LMR as a potentially useful prognostic marker in cervical cancer.

A biologically plausible explanation for these associations is that LMR captures the balance between anti-tumor immune activity and pro-tumor inflammation. Lymphocytes—particularly cytotoxic T cells and natural killer cells—contribute to immune surveillance and tumor cell killing (Van der Leun, Thommen & Schumacher, 2020; Downs-Canner et al., 2022). In contrast, circulating monocytes can differentiate into tumor-associated macrophages within the tumor microenvironment, promoting angiogenesis, immunosuppression, and metastasis through cytokines and growth factors (*e.g.*, IL-6, IL-10, and VEGF) (Giese, Hind & Huttenlocher, 2019). Thus, a low LMR may reflect relative lymphopenia and/or monocytosis, consistent with a more immunosuppressive and tumor-promoting milieu. In cervical carcinogenesis, persistent high-risk HPV infection and chronic inflammation are key contributors, which may further strengthen the relevance of systemic inflammatory markers such as LMR (Tomaziu-Todosia Anton et al., 2025).

Our subgroup analyses provide additional context for interpretation. In univariable analyses, the adverse association between low LMR and OS was generally observed across categories of age, histology, treatment, and LMR cut-off. In multivariable analyses, the association appeared attenuated in some subgroups, which may reflect differences in baseline risk profiles, treatment selection, and—importantly—the covariates included in adjustment models across studies. Because multivariable models were not uniform, residual confounding and between-study differences in adjustment sets could contribute to subgroup-level variability. Consequently, subgroup findings should be interpreted as hypothesis-generating rather than definitive evidence of effect modification.

Heterogeneity was low-to-moderate across the main pooled analyses, with the highest between-study heterogeneity observed in the univariable OS synthesis. Variation in study-specific LMR cut-offs likely contributed to these differences. In addition, the methods used to derive cut-off values varied across studies, including ROC-based, Youden-derived, and median-based approaches. Notably, meta-regression demonstrated that the LMR cut-off value was significantly associated with effect size in both OS-U and OS-M analyses, indicating that higher cut-off values were associated with smaller OS effect estimates. This finding supports the concern that cut-off heterogeneity may partly explain between-study variability and underscores the need for standardized, prospectively validated thresholds. While sensitivity analyses indicated that the overall conclusions were robust to omission of individual studies, future work should aim to standardize cut-off selection and evaluate whether clinically meaningful thresholds can be defined prospectively.

This study has several strengths. It synthesizes the currently available evidence on pretreatment LMR and survival in cervical cancer, distinguishes univariable from multivariable estimates, and incorporates subgroup, sensitivity, meta-regression, and publication-bias analyses. However, several limitations should be acknowledged. Most included studies were retrospective and geographically concentrated, predominantly in East Asia, which may affect external validity and limit generalizability to other populations and healthcare settings. Cut-off heterogeneity and non-uniform covariate adjustment may also have influenced pooled estimates. Importantly, the substantial heterogeneity in study-specific LMR cut-off selection may represent a form of selection bias, particularly when cut-offs are derived in a data-driven manner without formal validation. This methodological variation may have inflated pooled effect estimates and increased the risk of false-positive findings; therefore, the pooled prognostic magnitude of low pretreatment LMR should be interpreted with caution. In addition, the number of studies contributing multivariable PFS, and several subgroup analyses was limited, and publication-bias assessments for PFS should therefore be interpreted cautiously. Future studies should ideally adopt prospective multicenter designs, prespecified blood sampling windows before treatment, standardized laboratory measurement protocols, transparent and externally validated cut-off derivation strategies, and consistent adjustment for established clinicopathologic confounders such as FIGO stage, histology, tumor size, lymph node status, and treatment modality. Repeated LMR measurements during treatment and follow-up may further clarify whether dynamic changes provide additional prognostic value beyond a single pretreatment assessment.

## Conclusions

In conclusion, low pretreatment LMR is associated with poorer overall survival and progression-free survival in cervical cancer. Although the pooled findings were generally robust, the retrospective nature of the available evidence and the substantial heterogeneity in cut-off selection limit immediate clinical applicability. Prospective studies are needed to validate standardized and clinically meaningful cut-off values and to determine whether integrating LMR into established clinicopathologic risk models can improve pretreatment risk stratification.

##  Supplemental Information

10.7717/peerj.21337/supp-1 Supplemental Information 1 PRISMA checklist

10.7717/peerj.21337/supp-2 Supplemental Information 2 Detailed data

10.7717/peerj.21337/supp-3 Supplemental Information 3 Quality evaluation of the eligible studies with Newcastle–Ottawa scale

10.7717/peerj.21337/supp-4 Supplemental Information 4 Ultra-compact methodological extraction details of included cohorts

10.7717/peerj.21337/supp-5 Supplemental Information 5 Baseline characteristics of included studies

10.7717/peerj.21337/supp-6 Supplemental Information 6 Meta-regression plot, Funnel plot, publication bias and search strategy

## Additional Information and Declarations

### Data Availability

The following information was supplied regarding data availability: The data is available in the Supplementary File.
