# Supplementary material for: Prognostic value of lymphocyte to monocyte ratio for cervical cancer: a systematic review and meta-analysis
Source: PeerJ. 2026 May 27;14:e21337. doi: 10.7717/peerj.21337 (PMC13221991; doi:10.7717/peerj.21337)
Supplement: Supplemental Information 3 [file peerj-14-21337-s003.docx]

**Table S1** **Quality evaluation of the eligible studies with Newcastle–Ottawa scale.**

| Study | Selection | | | | Comparability | | | Outcome | |
| --- | --- | --- | --- | --- | --- | --- | --- | --- | --- |
|  | Representative-ness | Selection of non-exposed | Ascertainment of exposure | Outcome not present at start | Comparability on most important factors | Comparability on other risk factors | Assessment of outcome | Long enough follow-up (median≥1 year) | Adequacy (completeness) of follow-up |
| (Ayhan et al. 2022) | * | * | * | * | * | - | * | * | * |
| (Chao et al. 2020) | * | * | * | * | * | - | * | * | * |
| (Chen et al. 2015) | * | * | * | * | - | - | * | * | * |
| (Cheng et al. 2022) | * | * | * | - | - | - | * | * | * |
| (Deng et al. 2021) | * | * | * | * | - | - | * | * | * |
| (Gao et al. 2024) | * | * | * | * | - | - | * | * | * |
| (Guo et al. 2023a) | * | * | * | * | - | - | * | * | * |
| (Guo et al. 2023b) | * | * | * | * | * | - | * | * | * |
| (Hao et al. 2025) | * | * | * | * | - | - | * | * | * |
| (Huang et al. 2019) | * | * | * | * | * | - | * | * | * |
| (Jia et al. 2025) | * | * | * | * | * | - | * | * | * |
| (Koca et al. 2025) | * | * | * | * | - | - | * | * | * |
| (Kumar et al. 2024) | * | * | * | * | * | - | * | * | * |
| (Li et al. 2021) | * | * | * | * | * | - | * | * | * |
| (Chen et al. 2024) | * | * | * | * | * | - | * | * | * |
| (Liu et al. 2022) | * | * | * | * | * | - | * | * | * |
| (Wang et al. 2023) | * | * | * | * | * | - | * | * | * |
| (Xu et al. 2021) | * | * | * | * | * | - | * | * | * |

*indicates criterion met; - indicates significant of criterion not met.
