# Supplementary material for: Prognostic value of lymphocyte to monocyte ratio for cervical cancer: a systematic review and meta-analysis
Source: PeerJ. 2026 May 27;14:e21337. doi: 10.7717/peerj.21337 (PMC13221991; doi:10.7717/peerj.21337)
Supplement: Supplemental Information 4 [file peerj-14-21337-s004.docx]

**Table S2 Ultra-compact methodological extraction details of included cohorts.**

| Cohort | Marker → LMR cutoff | Cutoff method / validation | Blood timing / serial assessment | Multivariable handling / final factor(s) | Cohort note |
| --- | --- | --- | --- | --- | --- |
| Ayhan, S.(2022) | M/L → 3.66 | ROC; validation NR | 8 ± 6 d before surgery; pretreatment only | Stepwise Cox; final: FIGO, NM/L; no adjusted M/L retained | Single-center surgical cohort |
| Chao, B.(2020)(1) | MLR → 3.45 | Youden; primary-derived | ≤1 wk preop; postop 4–8 wk; serial | Forward stepwise Cox; inflammatory markers compared; SIRI emphasized | Primary cohort |
| Chao, B.(2020)(2) | MLR → 3.45 | Applied from primary cohort; within-article validation | Same article-level timing; serial | Same article-level model strategy | Validation cohort, different hospital |
| Chen, L.(2015) | LMR → 2.87 | ROC; validation NR | Pretreatment, within 2 d after admission; pretreatment only | Multivariable Cox; LMR remained significant for RFS/OS | Direct LMR study |
| Cheng, M.(2022) | MLR → 3.45 | ROC; validation NR | Pretreatment before immunotherapy; pretreatment only | PFS model only; final: histology, NLR, LDH | Metastatic/recurrent immunotherapy cohort; no OS |
| Deng, Q.(2021)(1) | MLR → 3.70 | ROC; within-article validation only | ≤1 wk before surgery; pretreatment only | Reverse stepwise Cox; final: LVSI, FIGO, MPV/PC | Primary cohort |
| Deng, Q.(2021)(2) | MLR → 3.70 | Applied from primary cohort; within-article validation only | ≤1 wk before surgery; pretreatment only | Same article-level model strategy | Validation cohort, different hospital |
| Gao, T.(2024) | MLR → 3.85 | Median-based / NR | Preoperative, exact window NR; pretreatment only | Multivariable Cox; adjusted MLR significant | Stage IIIC1p-only cohort |
| Guo, H.(2023) | LMR → 4.04 | NR / NR | Pretreatment, exact window NR; pretreatment only | Final model emphasized IIIC1r, SII, sarcopenia, SAI, VAI | Body composition + inflammation model |

**Table S2 (continued) Ultra-compact methodological extraction details of included cohorts.**

| Study/cohort | Marker → LMR cutoff | Cutoff method / validation | Blood timing / serial assessment | Multivariable handling / final factor(s) | Cohort note |
| --- | --- | --- | --- | --- | --- |
| Guo, J.(2023) | MLR → 4.17 | ROC; validation NR | Preop/pretreatment, exact window NR; pretreatment only | OPNI, not MLR, was independent | Early-stage poorly/moderately differentiated CSCC |
| Hao, F.(2025) | LMR → 3.50 | Median-based; validation NR | Pretreatment, exact window NR; pretreatment only | Final: FIGO stage, miscarriage history | Two-center SCC radiotherapy cohort |
| Huang, H.(2019)(1) | MLR → 3.85 | Primary-derived; within-article validation only | Preoperative, exact window NR; pretreatment only | Final inflammatory predictor: SII, not MLR | Primary cohort |
| Huang, H.(2019)(2) | MLR → 3.85 | Applied from primary cohort; within-article validation only | Preoperative, exact window NR; pretreatment only | Same article-level model strategy | Validation cohort |
| Jia, S.(2025) | LMR → 6.20 | X-tile (based on PFS); validation NR | Pretreatment hematology; pretreatment only | PNI dominant in multivariable models; LMR not retained | FIGO III–IVA SCC HR-LACC, curative CRT |
| Koca, T.(2025) | MLR → 3.33 | NR / NR | Within 1 mo before treatment; pretreatment only | Final: PIV for PFS; CRP/ECOG for OS | Definitive CCRT cohort |
| Kumar, A.(2024) | LMR → 4.41 | Median-based grouping; validation NR | Pretreatment baseline hematology; pretreatment only | Multivariable Cox; LMR modeled as continuous | Large RT/CRT cohort |
| Li, Y.-X.(2021) | MLR → 3.85 | Median-based; no validation cohort | Pretreatment routine blood test; pretreatment only | Adjusted MLR significant for OS and PFS | Stage IIB-only cohort |
| Ling-Yu, J.(2024) | MLR → 3.85 | ROC; validation NR | Pretreatment; post-tx 14 d–8 wk (median 19 d); serial | Prognostic evaluation included PNI/NLR/MLR/PLR; no clearly separated adjusted MLR model | Multi-institutional radiochemotherapy cohort |

**Table S2 (continued) Ultra-compact methodological extraction details of included cohorts.**

| Study/cohort | Marker → LMR cutoff | Cutoff method / validation | Blood timing / serial assessment | Multivariable handling / final factor(s) | Cohort note |
| --- | --- | --- | --- | --- | --- |
| Liu, P.(2022)(1) | LMR → 12.25 (survival); 6.2917 (pCR) | ROC; temporal within-article validation only | Within 7 d before NACT; pretreatment only | Cox for OS/PFS; final emphasis on SII rather than LMR | Training cohort |
| Liu, P.(2022)(2) | LMR → 12.25 (survival); 6.2917 (pCR) | Applied from training cohort; temporal within-article validation only | Within 7 d before NACT; pretreatment only | Same article-level model strategy | Validation cohort |
| Wang, H.-B.(2023) | MLR → 2.44 | NR / NR | Before RT; pretreatment only | Adjusted MLR significant for OS and PFS | Stage IIB–III RT cohort |
| Xu, M.(2021) | LMR → 4.10 | X-tile; validation NR | Preoperative, exact window NR; pretreatment only | Final: FIGO, LVSI, LN metastasis, albumin, LMR | Direct LMR study; SIS-focused article |

Abbreviations: LMR, lymphocyte-to-monocyte ratio; MLR, monocyte-to-lymphocyte ratio; M/L, monocyte-to-lymphocyte ratio; NR, not reported; preop, preoperative; postop, postoperative; wk, week; mo, month; d, day; tx, treatment; RT, radiotherapy; CRT, chemoradiotherapy; CCRT, concurrent chemoradiotherapy; NACT, neoadjuvant chemotherapy; LVSI, lymphovascular space invasion; LN, lymph node; SCC, squamous cell carcinoma; CSCC, cervical squamous cell carcinoma; PIV, pan-immune-inflammation value.

Note: For studies reporting MLR or M/L instead of LMR, the reported cut-off was harmonized to the LMR scale for methodological comparison.
