# Supplementary material for: Prognostic value of lymphocyte to monocyte ratio for cervical cancer: a systematic review and meta-analysis
Source: PeerJ. 2026 May 27;14:e21337. doi: 10.7717/peerj.21337 (PMC13221991; doi:10.7717/peerj.21337)
Supplement: Supplemental Information 5 [file peerj-14-21337-s005.docx]

**Table S3** Baseline characteristics of included studies (References).

| Author | Year | region | Sample size | Study Period | Mean/median age | FIGO Stage | Treatment | Histological Type | Follow-up(month) Median(range) | LMR | Survival outcome | Survival analysis | NOS |  |
| --- | --- | --- | --- | --- | --- | --- | --- | --- | --- | --- | --- | --- | --- | --- |
|  |  |  |  |  |  |  |  |  |  |  |  |  |  |  |
| Koca, T. (Koca et al. 2025) | 2025 | Turkey | 90 | 2014.1-2023.4 | NA | I-IV | CRT | SCC/AC/others | 36.2(4.9–117.4) | 3.33 | OS/PFS | Univariate/Multivariate | 7 |  |
| Jia, S.(Jia et al. 2025) | 2025 | China | 157 | 2016.11-2024.4 | 55(26–88) | IIIB-IVA | CRT | SCC | 35(3-96) | 6.2 | OS/PFS | Univariate | 8 |  |
| Hao, F.(Hao et al. 2025) | 2025 | China | 99 | 2017.4-2022.3 | 60(35-86) | I-IV | RT | SCC | 33 | 3.5 | OS | Univariate | 7 |  |
| Ling-Yu, J.(Chen et al. 2024) | 2024 | China | 138 | 2016-2021 | 60.1 ± 14.1 | IB-IVA | CRT | SCC/AC/others | 33.8(7.2-79.3) | 3.85 | OS/PFS | Univariate | 7 |  |
| Kumar, A.(Kumar et al. 2024) | 2024 | India | 1051 | 2003-2017 | 50(24-66) | IB2-IVA | CRT | SCC/AC/others | 69(3-203) | 4.41 | OS/DFS | Univariate | 8 |  |
| Wang, H.-B.(Wang et al. 2023) | 2023 | China | 178 | 2013.9-2015.9 | 53.85 | II-III | RT | SCC/AC | NA | 2.44 | OS/PFS | Univariate/Multivariate | 8 |  |
| Gao, T.(Gao et al. 2024) | 2024 | China | 186 | 2012.3-2022.3 | 47.11 ± 9.15 | IIIC1p | radical operation+CRT | SCC/AC/others | 51.1(30-91) | 3.85 | OS | Univariate/Multivariate | 7 |  |
| Guo, J.(Guo et al. 2023b) | 2023 | China | 109 | 2014.5-2017.12 | 53.95 ± 9.6 | I-IIA | radical operation | SCC | NA | 4.17 | OS | Univariate | 8 |  |
| Guo, H.(Guo et al. 2023a) | 2023 | China | 196 | 2016.1-2019.6 | 52 | IIB-IIIC | CRT | SCC/AC | 64.8 | 4.04 | OS | Univariate/Multivariate | 7 |  |
| Liu, P.(1)(Liu et al. 2022) | 2022 | China | 133 | 2005.11-2012.11 | 51(29-69) | IB2-IIB | NACT+radical operation | SCC | 98(4-156) | 12.25 | OS/PFS | Univariate | 8 |  |
| Liu, P.(2)(Liu et al. 2022) | 2022 | China | 77 | 2013.1-2014.11 | 48(30-64) | IB2-IIB | NACT+radical operation | SCC | 84.6(13-106) | 12.25 | OS | Univariate | 8 |  |
| Cheng, M.(Cheng et al. 2022) | 2022 | China | 70 | 2019.6-2021.4 | 51(29–77) | NA | Combination Immunotherapy | SCC/AC/others | NA | 3.45 | PFS | Univariate | 6 |  |
| Ayhan, S.(Ayhan et al. 2022) | 2022 | Turkey | 163 | 2008.2-2018.10 | 49(32-78) | IA-IIIC | radical operation ±RT/CT | SCC/AC/others | NA | 3.66 | OS/DFS | Univariate | 8 |  |
| Xu, M.(Xu et al. 2021) | 2021 | China | 264 | 2014.1-2017.12 | 47(24-71) | IB-IIA | radical operation | SCC/AC/others | 44(3-72) | 4.1 | OS | Univariate/Multivariate | 8 |  |
| Li, Y.-X.(Li et al. 2021) | 2021 | China | 260 | 2011.3-2016.10 | 51(28-74) | IIB | NACT ±RT | SCC/AC/others | 47.3(2.63-101.4) | 3.85 | OS/PFS | Univariate/Multivariate | 8 |  |
| Deng, Q.(1)(Deng et al. 2021) | 2021 | China | 283 | 2009-2017 | 47(24-84) | IA-IIA | radical operation±RT | SCC | 72(4-129) | 3.7 | OS | Univariate/Multivariate | 7 |  |
| Deng, Q.(2)(Deng et al. 2021) | 2021 | China | 125 | NA | 43(24-67) | IA-IIA | radical operation±RT | SCC | 47(3-120) | 3.7 | OS | Univariate/Multivariate | 7 |  |
| Chao, B.(1)(Chao et al. 2020) | 2020 | China | 441 | 2009-2018 | 42(28–79) | IA-IIA | radical operation ±RT/CT | SCC | 67(6-129) | 3.45 | OS | Univariate/Multivariate | 8 |  |
| Chao, B.(2)(Chao et al. 2020) | 2020 | China | 164 | NA | NA | IA-IIA | radical operation ±RT/CT | SCC | NA | 3.45 | OS | Univariate/Multivariate | 8 |  |
| Huang, H.(1)(Huang et al. 2019) | 2019 | China | 328 | 2006-2015 | 45(22-86) | IA-IIA | radical operation | SCC | 47(3-129) | 3.85 | OS | Univariate/Multivariate | 8 |  |
| Huang, H.(2)(Huang et al. 2019) | 2019 | China | 130 | NA | 44(23-68) | IA-IIA | radical operation | SCC | 47(3-120) | 3.85 | OS | Univariate/Multivariate | 8 |  |
| Chen, L.(Chen et al. 2015) | 2015 | China | 485 | 2004.1-2008.12 | 45 | IB1-IIA | radical operation ±RT/CT | SCC/AC/others | 75(10-118) | 2.87 | OS/RFS | Univariate/Multivariate | 7 |  |

CRT chemoradiotherapy, RT Radiotherapy, NACT neoadjuvant chemotherapy, CT chemotherapy, SCC Squamous cell carcinoma, AC Adenocarcinoma, OS overall survival, PFS progression-free survival, LMR LMR lymphocyte-to-monocyte ratio, NOS Newcastle–Ottawa Scale, NA Not available.

(1)(2) Two independent cohorts in the same study.

**References**

Ayhan S, Akar S, Kar İ, Turan AT, Türkmen O, Kiliç F, Aytekin O, Ersak B, Ceylan Ö, Moraloğlu Tekin Ö, and Kimyon Comert G. 2022. Prognostic value of systemic inflammatory response markers in cervical cancer. *J Obstet Gynaecol* 42:2411-2419. 10.1080/01443615.2022.2069482

Chao B, Ju X, Zhang L, Xu X, and Zhao Y. 2020. A Novel Prognostic Marker Systemic Inflammation Response Index (SIRI) for Operable Cervical Cancer Patients. *Frontiers in Oncology* 10. 10.3389/fonc.2020.00766

Chen JLY, Huang CY, Shih IL, Liou YM, Tai YJ, Chiang YC, and Kuo CY. 2024. Prognostic nutritional index and neutrophil-lymphocyte ratio predict toxicities and prognosis in patients with cervical cancer treated with curative radiochemotherapy. *Journal of the Formosan Medical Association* 123:671-678. 10.1016/j.jfma.2023.10.022

Chen L, Zhang F, Sheng XG, and Zhang SQ. 2015. Decreased pretreatment lymphocyte/monocyte ratio is associated with poor prognosis in stage Ib1-IIa cervical cancer patients who undergo radical surgery. *Onco Targets Ther* 8:1355-1362. 10.2147/ott.S82174

Cheng M, Li G, Liu Z, Yang Q, and Jiang Y. 2022. Pretreatment Neutrophil-to-Lymphocyte Ratio and Lactate Dehydrogenase Predict the Prognosis of Metastatic Cervical Cancer Treated with Combination Immunotherapy. *Journal of Oncology* 2022. 10.1155/2022/1828473

Deng Q, Long Q, Liu Y, Yang Z, Du Y, and Chen X. 2021. Prognostic value of preoperative peripheral blood mean platelet volume/platelet count ratio (MPV/PC) in patients with resectable cervical cancer. *Bmc Cancer* 21. 10.1186/s12885-021-09016-8

Gao T, Yang Z, Wei L, Tang X, Ma S, Jiang L, Zhang Y, and Wu F. 2024. Prognostic analysis of stage IIIC1p cervical cancer patients. *Frontiers in Oncology* 14. 10.3389/fonc.2024.1362281

Guo H, Feng S, Yin Y, and Li D. 2023a. Prognostic value of body composition and systemic inflammatory markers in patients with locally advanced cervical cancer following chemoradiotherapy. *Annals of Oncology* 34:S1591. 10.1016/j.annonc.2023.10.425

Guo J, Lv W, Wang Z, Shang Y, Yang F, Zhang X, Xiao K, Zhang S, Pan X, Han Y, Zong L, and Hu W. 2023b. Prognostic Value of Inflammatory and Nutritional Markers for Patients With Early-Stage Poorly-to Moderately-Differentiated Cervical Squamous Cell Carcinoma. *Cancer Control* 30. 10.1177/10732748221148913

Hao F, Xiao N, Wang M, Sang H, Luo J, and Song J. 2025. Clinical Prognostic Factors for Overall Survival in Patients Undergoing Radical Radiotherapy for Cervical Cancer. *International Journal of Women's Health* 17:3347-3359. 10.2147/IJWH.S543995

Huang H, Liu Q, Zhu L, Zhang Y, Lu X, Wu Y, and Liu L. 2019. Prognostic Value of Preoperative Systemic Immune-Inflammation Index in Patients with Cervical Cancer. *Scientific Reports* 9. 10.1038/s41598-019-39150-0

Jia S-z, Yang X-j, Yang D, Wang R, Yang X, Huang M-n, and An J-s. 2025. Low pretreatment prognostic nutritional index predicts unfavorable survival in stage III-IVA squamous cervical cancer undergoing chemoradiotherapy. *Bmc Cancer* 25. 10.1186/s12885-025-13752-6

Koca T, Gocen Vardar N, Aksoy RA, and Korcum AF. 2025. Comprehensive Evaluation of Inflammatory Biomarkers in Cervical Cancer Treated with Chemoradiotherapy. *Curr Oncol* 32. 10.3390/curroncol32010039

Kumar A, Gurram L, Naga Ch P, Nayak P, Mulye G, Chopra S, Engineer R, Shrivastava SK, Gupta S, Ghosh J, Gulia S, Agarwal JP, and Mahantshetty U. 2024. Correlation of Hematological Parameters With Clinical Outcomes in Cervical Cancer Patients Treated With Radical Radio(chemo)therapy: A Retrospective Study. *Int J Radiat Oncol Biol Phys* 118:182-191. 10.1016/j.ijrobp.2023.07.022

Li YX, Chang JY, He MY, Wang HR, Luo DQ, Li FH, Li JH, and Ran L. 2021. Neutrophil-to-Lymphocyte Ratio (NLR) and Monocyte-to-Lymphocyte Ratio (MLR) Predict Clinical Outcome in Patients with Stage IIB Cervical Cancer. *J Oncol* 2021:2939162. 10.1155/2021/2939162

Liu P, Jiang Y, Zheng X, Pan B, Xiang H, and Zheng M. 2022. Pretreatment Systemic Immune-Inflammation Index Can Predict Response to Neoadjuvant Chemotherapy in Cervical Cancer at Stages IB2-IIB. *Pathology & Oncology Research* 28. 10.3389/pore.2022.1610294

Wang H-B, Xu X-T, Tian M-X, Ding C-C, Tang J, Qian Y, and Jin X. 2023. Prognostic values of the prognostic nutritional index, geriatric nutritional risk index, and systemic inflammatory indexes in patients with stage IIB-III cervical cancer receiving radiotherapy. *Frontiers in Nutrition* 10. 10.3389/fnut.2023.1000326

Xu M, Wu Q, Cai L, Sun X, Xie X, and Sun P. 2021. Systemic Inflammatory Score predicts Overall Survival in patients with Cervical Cancer. *Journal of Cancer* 12:3671-3677. 10.7150/jca.56170
