# Supplementary material for: Prognostic value of lymphocyte to monocyte ratio for cervical cancer: a systematic review and meta-analysis
Source: PeerJ. 2026 May 27;14:e21337. doi: 10.7717/peerj.21337 (PMC13221991; doi:10.7717/peerj.21337)
Supplement: Supplemental Information 6 [file peerj-14-21337-s006.pdf]

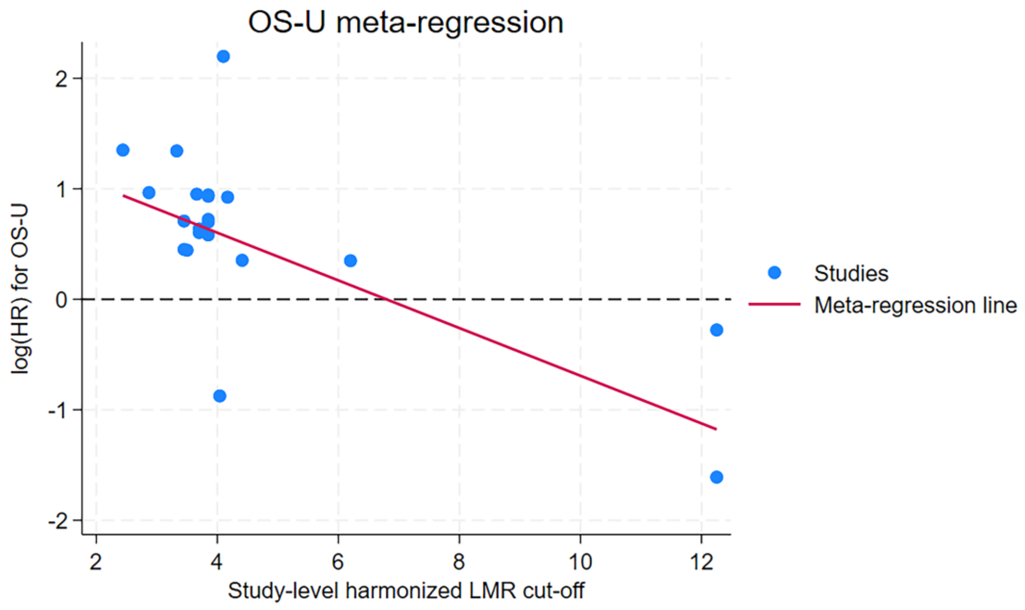

Figure S1 Meta-regression of univariable OS effect estimates against the study-level harmonized LMR cut-off.

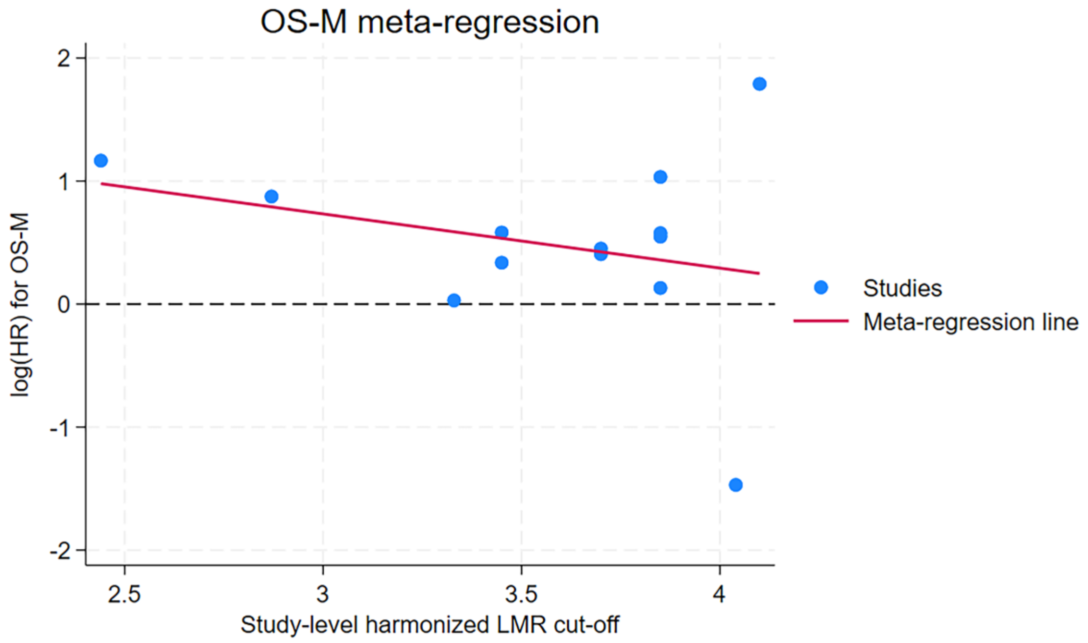

Figure S2 Meta-regression of multivariable OS effect estimates against the study-level harmonized LMR cut-off.

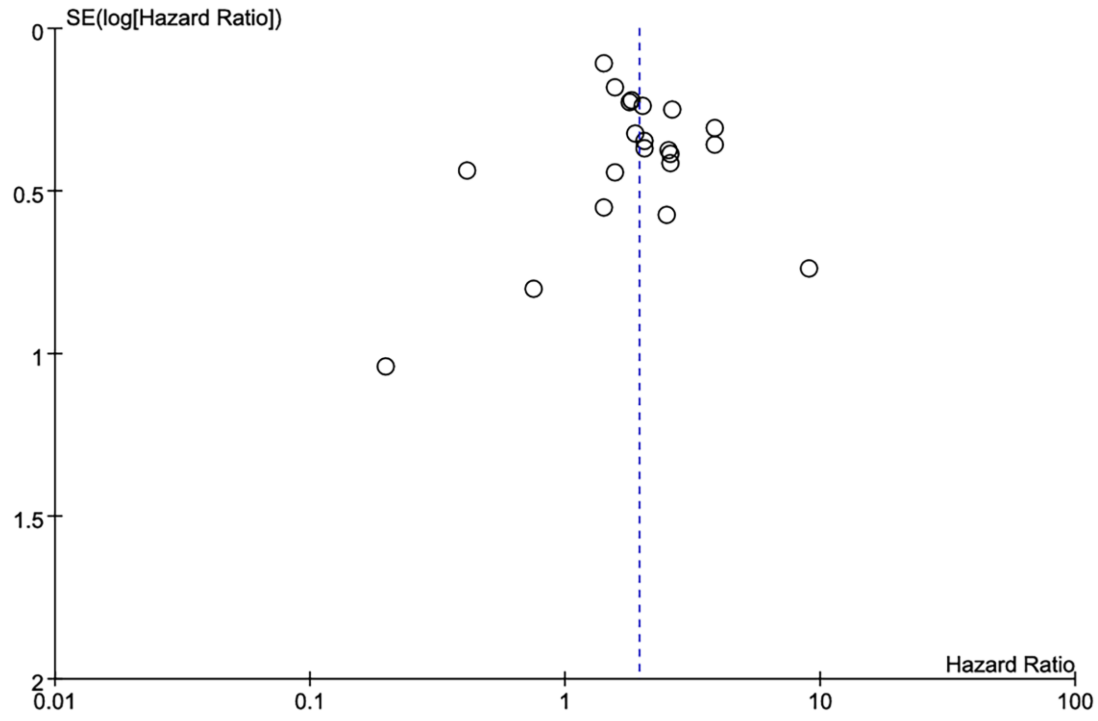

Figure S3 Funnel plot for univariable overall survival (OS) analysis.

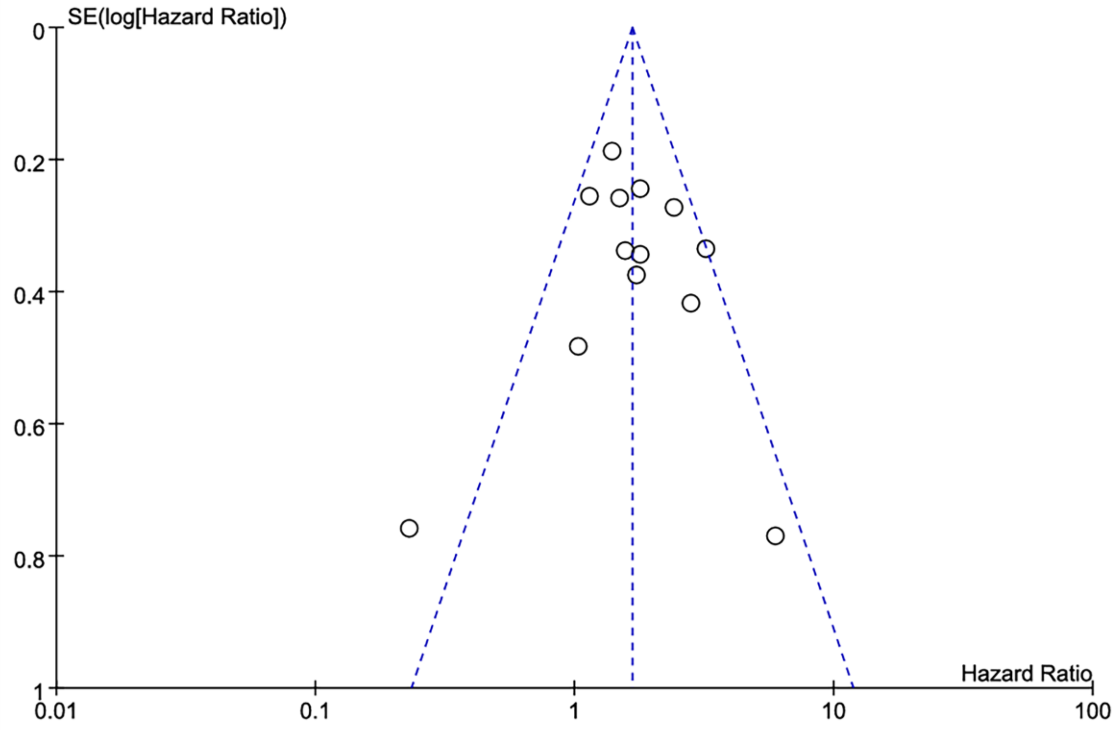

Figure S4 Funnel plot for multivariable overall survival (OS) analysis.

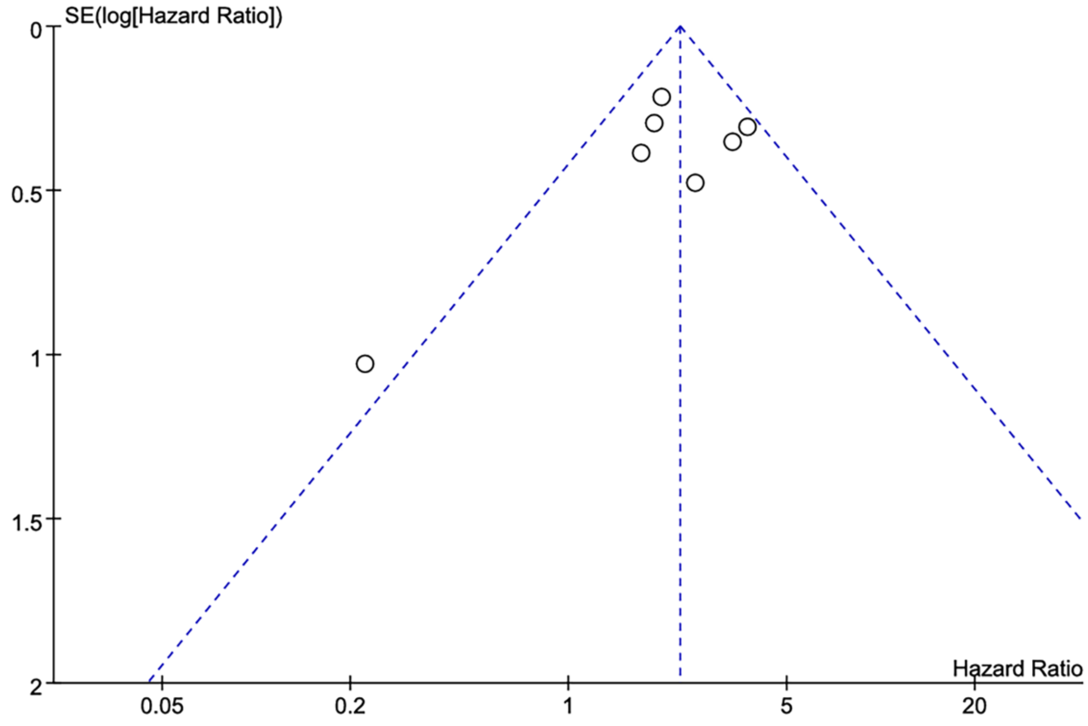

Figure S5 Funnel plot for univariable progression-free survival (PFS) analysis.

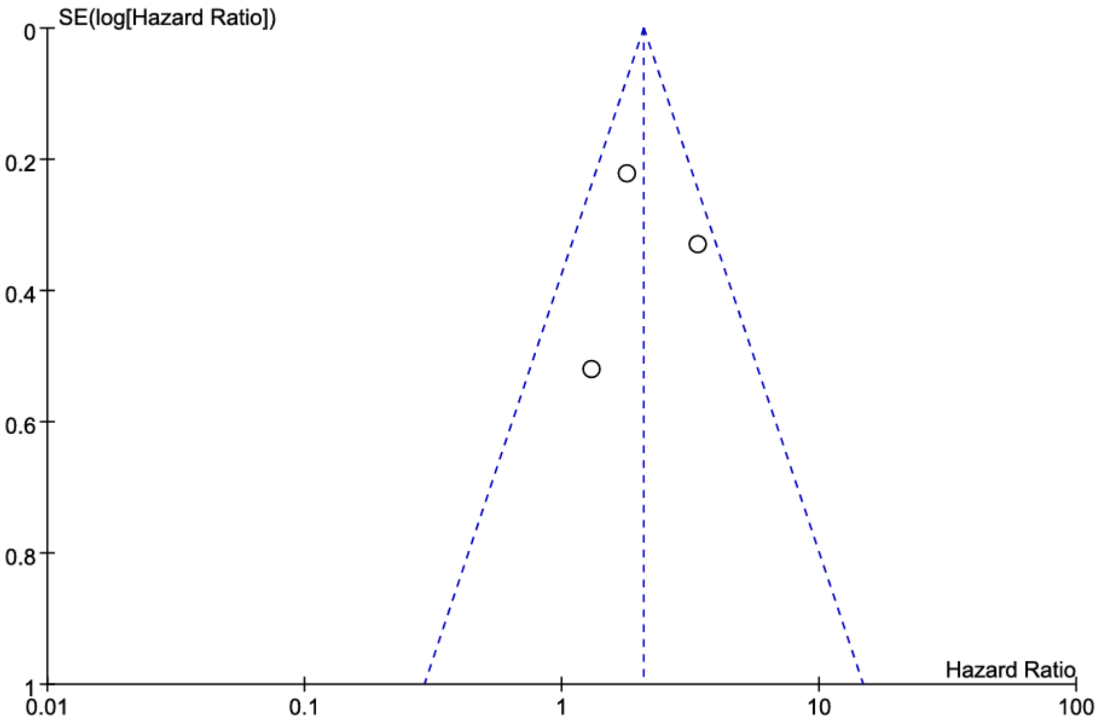

Figure S6 Funnel plot for multivariable progression-free survival (PFS) analysis.

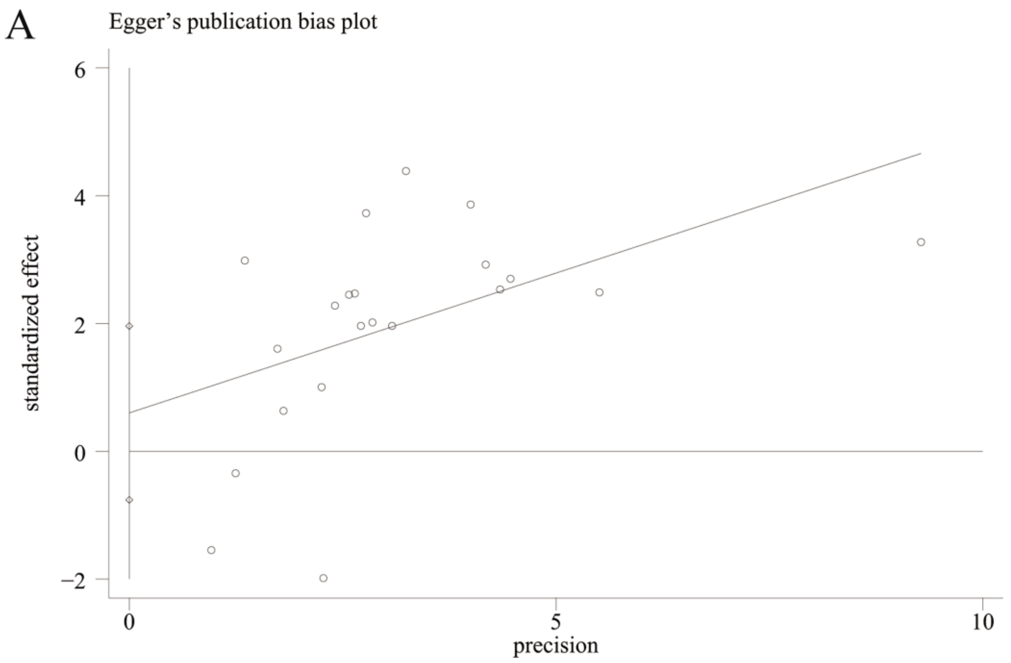
A
Egger's publication bias plot
standardized effect
precision
6
4
2
0
−2
0
5
10

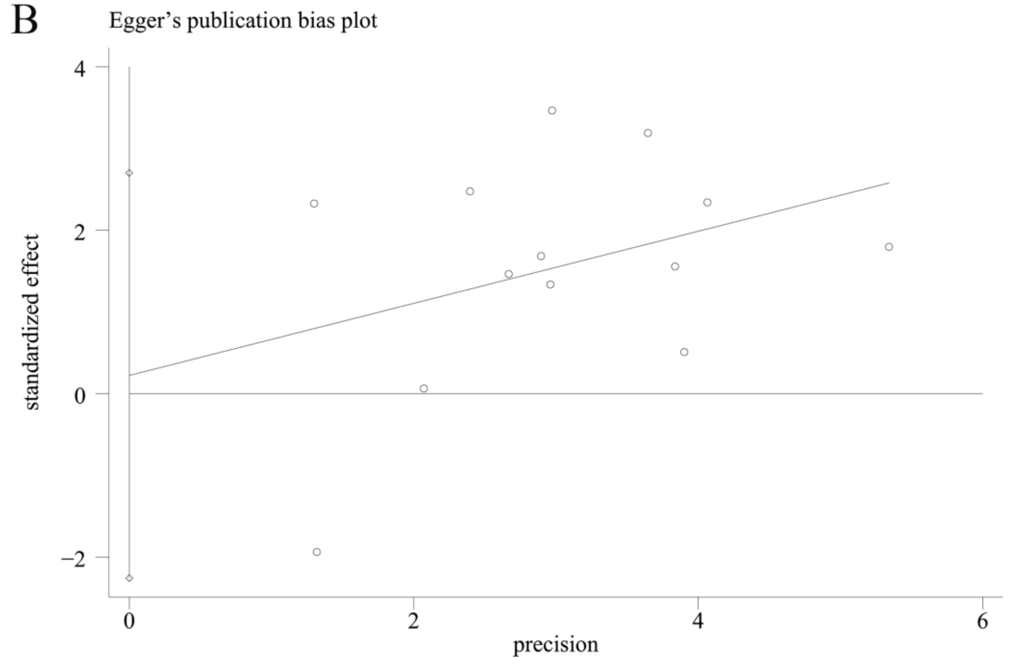
B
Egger's publication bias plot
standardized effect
precision
4
2
0
−2
0
2
4
6

C

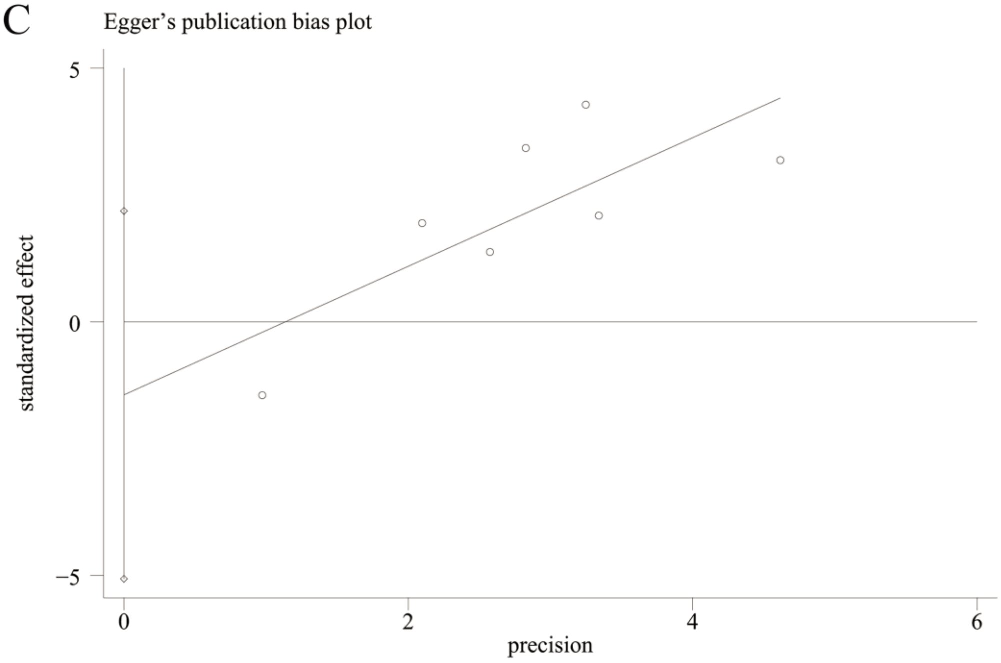

D

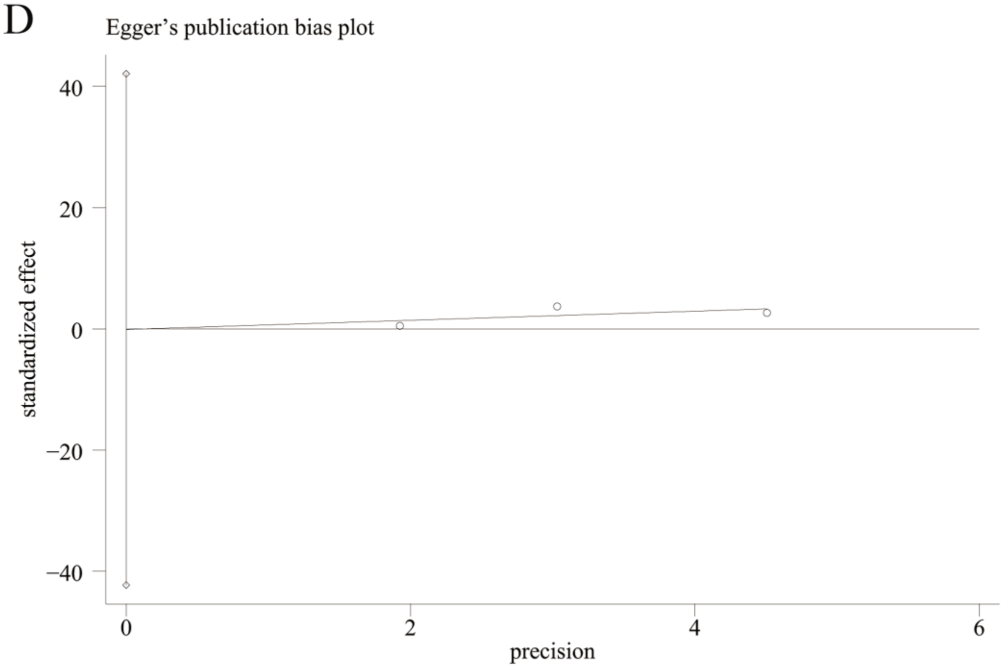

Figure S7 Egger's regression plots for publication bias in OS and PFS analyses.

(A) OS-U, P = 0.368; (B) OS-M, P = 0.778; (C) PFS-U, P = 0.353; (D) PFS-M, P = 0.959.

Appendix (Retrieval database deadline-November 14, 2025)

PubMed-81

((ratio) AND ((((((((Lymphocytes) OR (Lymphocyte)) OR (Lymphoid Cells)) OR (Cell, Lymphoid)) OR (Cells, Lymphoid)) OR (Lymphoid Cell)) OR ("Lymphocytes"[Mesh])) AND (((Monocytes) OR (Monocyte)) OR ("Monocytes"[Mesh])))) AND ((((((((((((((((((((((((Uterine Cervical Neoplasms) OR (Cervical Neoplasm, Uterine)) OR (Neoplasm, Uterine Cervical)) OR (Uterine Cervical Neoplasm)) OR (Neoplasms, Cervix)) OR (Cervix Neoplasm)) OR (Neoplasm, Cervix)) OR (Cervix Neoplasms)) OR (Cervical Neoplasms)) OR (Cervical Neoplasm)) OR (Neoplasms, Cervical)) OR (Cancer of the Uterine Cervix)) OR (Cancer of Cervix)) OR (Cancer of the Cervix)) OR (Cervix Cancer)) OR (Cancer, Cervix)) OR (Uterine Cervical Cancer)) OR (Cancer, Uterine Cervical)) OR (Cervical Cancer, Uterine)) OR (Uterine Cervical Cancers)) OR (Cervical Cancer)) OR (Cancer, Cervical)) OR (Cervical Cancers)) OR ("Uterine Cervical Neoplasms"[Mesh]))

OVID-Web of Science-107, Embase-180, Cochrane-3

((ratio) AND ((((((((Lymphocytes) OR (Lymphocyte)) OR (Lymphoid Cells)) OR (Cell, Lymphoid)) OR (Cells, Lymphoid)) OR (Lymphoid Cell)) OR (Lymphocytes)) AND (((Monocytes) OR (Monocyte)) OR (Monocytes)))) AND ((((((((((((((((((((((((Uterine Cervical Neoplasms) OR (Cervical Neoplasm, Uterine)) OR (Neoplasm, Uterine Cervical)) OR (Uterine Cervical Neoplasm)) OR (Neoplasms, Cervix)) OR (Cervix Neoplasm)) OR (Neoplasm, Cervix)) OR (Cervix Neoplasms)) OR (Cervical Neoplasms)) OR (Cervical Neoplasm)) OR (Neoplasms, Cervical)) OR (Cancer of the Uterine Cervix)) OR (Cancer of Cervix)) OR (Cancer of the Cervix)) OR (Cervix Cancer)) OR (Cancer, Cervix)) OR (Uterine Cervical Cancer)) OR (Cancer, Uterine Cervical)) OR (Cervical Cancer, Uterine)) OR (Uterine Cervical Cancers)) OR (Cervical Cancer)) OR (Cancer, Cervical)) OR (Cervical Cancers)) OR (Uterine Cervical Neoplasms))
